# Electroacupuncture exerts neuroprotective effects and alters gut microbiota in a MPTP-induced mouse model of Parkinson’s disease

**DOI:** 10.3389/fnins.2025.1702912

**Published:** 2026-01-14

**Authors:** Xi-Chen Wu, Yi-Yue Dong, Yu-Chen Ying, Guang-Yan Chen, Xi Wang, Qian Fan, Ping Yin, Yue-Lai Chen

**Affiliations:** 1 LongHua Hospital, Shanghai University of Traditional Chinese Medicine, Shanghai, China; 2 Gansu Provincial People’s Hospital, Jangsu University, Gansu, China

**Keywords:** 16S rRNA, electroacupuncture, gut microbiota, MPTP, Parkinson’s disease

## Abstract

**Objective:**

To investigate the therapeutic mechanism of electroacupuncture (EA) in a mouse model of Parkinson’s disease (PD) induced by 1-Methyl-4-phenyl-1,2,3,6-tetrahydropyridine (MPTP).

**Methods:**

Motor functions were evaluated using open field test and pole tests. Tyrosine hydroxylase (TH) expression in the substantia nigra and striatum was detected by immunohistochemistry. Intestinal barrier integrity was assessed via immunofluorescence staining of tight junction proteins ZO-1 and Occludin. Gut microbiota composition was analyzed by 16S rRNA sequencing.

**Results:**

EA treatment significantly improved motor deficits, restored TH expression in nigrostriatal regions, and enhanced colonic ZO-1 and Occludin levels. EA reversed MPTP-induced dysbiosis, notably normalizing the abundances of Dubosiella, Lactobacillus, Enterococcus, Desulfovibrio, Bacteroides, Allobaculum, and Parasutterella. Microbial co-occurrence network analysis revealed that EA simplified hyperconnected interactions and improved network stability.

**Conclusion:**

EA treatment attenuated PD progression, which was associated with the remodeling of gut microbiota structure and restoration of microbial network stability. The concomitant protection of dopaminergic function suggests a potential link mediated by the gut–brain axis.

## Introduction

Parkinson’s disease (PD) manifests clinically as a triad of core motor impairments: bradykinesia, resting tremor, and dyskinesia. Pathologically, this syndrome arises from progressive degeneration of substantia nigra dopaminergic neurons, concomitant with intraneuronal accumulation of α-synuclein (α-SYN) aggregates known as Lewy bodies (Gibb and Lees, 1988). Epidemiologically, PD exhibits a global incidence ranging between 8 and 18 cases per 100,000 person-years (Al-Kuraishy et al., 2023), with prevalence exceeding 2% in populations aged ≥ 65 years and escalating with advancing age (Hong et al., 2022). Current therapeutic strategies comprise pharmacological and neurosurgical modalities. Pharmacological interventions include: Dopamine receptor agonists, Monoamine oxidase B inhibitors (MAO-BIs), Catechol-O-methyltransferase inhibitors, Anticholinergic agents, N-methyl-D-aspartate (NMDA) receptor antagonists (Wu et al., 2025). Although effective for initial dyskinesia management, prolonged pharmacotherapy frequently induces motor fluctuations and paradoxically exacerbates dyskinetic symptoms in advanced PD (Stevenson, 1997; Waller et al., 2021). Neurosurgical approaches like deep brain stimulation demonstrate symptomatic efficacy but encounter barriers to widespread implementation due to technical complexity, substantial costs, and procedure-related complications (Serva et al., 2022).

Gastrointestinal (GI) manifestations, notably constipation, frequently precede motor dysfunction by decades in PD patients (Bloem et al., 2021). Emerging evidence indicates that pathological accumulation of misfolded α-SYN originates in the enteric nervous system, preceding central nervous system (CNS) symptom onset by years (Schapira et al., 2017). Furthermore, substantial research supports the gastrointestinal tract as a potential site of PD initiation, highlighting its pivotal role in disease progression (Abbott et al., 2001). The gut microbiota, colonizing the GI tract, is indispensable for host-microbial communication (Ley et al., 2006). Compelling evidence demonstrates microbial influence on bidirectional gut-brain axis signaling, wherein gut microbes contribute to PD pathogenesis through modulation of metabolites, gastrointestinal epithelial barrier integrity, and immune function (Jiang et al., 2017). Concomitant dysbiosis in bacterial composition and functionality correlates with PD pathology. Notably, PD patients with GI symptoms exhibit significant alterations in gut microbiota composition and metabolic activity compared to healthy controls (Hill-Burns et al., 2017). Crucially, fecal microbiota transplantation from PD patients into germ-free mice exacerbates motor deficits—an effect reversible by antibiotic treatment—establishing causal involvement of gut microbes in PD pathogenesis (Sampson et al., 2016). Clinical studies further confirm that specific probiotic regimens variably ameliorate PD symptoms. Collectively, these findings underscore the gut microbiota’s instrumental role in PD development.

Acupuncture, an ancient therapeutic modality rooted in traditional Chinese medicine, exerts regulatory effects on physiological processes through targeted stimulation of meridian-associated acupoints. Contemporary neurobiological research has demonstrated that acupuncture stimulation activates peripheral neural pathways, triggering reflex responses that propagate sensory signals through spinal cord pathways to supraspinal centers, ultimately inducing physiological modulation via autonomic nervous system regulation (Liu et al., 2021). Accumulating clinical and experimental evidence indicates that this neural regulatory mechanism may offer therapeutic benefits for multiple central nervous system pathologies, particularly in stroke rehabilitation (Choi et al., 2022), pain management (Lam et al., 2022), spinal cord injury recovery (Tan et al., 2022), and mood disorder treatment (Kim et al., 2022). Given the limitations of pharmacological and surgical interventions, acupuncture has been increasingly utilized as a complementary and alternative therapeutic approach for PD management. Clinical studies have demonstrated that acupuncture can ameliorate both motor and non-motor symptoms in PD patients while modulating gut microbiota composition (Zhang J. et al., 2023). Preclinical investigations have further confirmed that acupuncture intervention promotes normalization of gut microbial dysbiosis and suppresses neuroinflammation in the substantia nigra (SN) (Jang et al., 2020). Additional research has reported that electroacupuncture (EA) alleviates behavioral deficits in PD model mice by regulating gut microbiota and inhibiting lipid peroxidation in the SN pars compacta (SNpc). However, the mechanistic link between acupuncture-mediated gut microbiota modulation and subsequent improvement in central nervous system pathology remains unclear (Hu et al., 2024). Therefore, this study employs 16S rRNA sequencing to elucidate how EA improves motor function in MPTP-induced PD mice through its effects on gut microbiota and associated metabolic alterations.

## Materials and method

### Animals

Male C57BL/6 mice (8 weeks old; body weight 23–25 g) were sourced from Shanghai Xipur-Biak Co., Ltd. (China). Animals were housed under specific pathogen-free (SPF) conditions with environmental controls: temperature 21–23°C, relative humidity 50–60%, and *ad libitum* access to standard rodent chow. All experimental procedures were approved by the Institutional Animal Care and Use Committee (IACUC) of Longhua Hospital (Approval No. LHERWA-25038) and strictly adhered to internationally recognized guidelines for laboratory animal welfare.

### MPTP-induced PD mouse model

Following a 7-day acclimatization under standardized housing, mice were randomly allocated to three groups: (1) The control group received daily intraperitoneal (i.p.) injections of sterile saline, volume-matched to the 1-Methyl-4-phenyl-1,2,3,6-tetrahydropyridine (MPTP) group; (2) The MPTP model group received i.p. MPTP (30 mg/kg/day dissolved in 0.9% saline) for 5 consecutive days; (3) The EA intervention group, established following MPTP induction, underwent treatment targeting acupoints GV20 and GV14. EA was administered using an SDZ-V device (Huatuo, China) with parameters set at 100 Hz, 0.2–1 mA intensity, and 20 min daily sessions, delivered 5 days/week over 14 days. Both the MPTP and EA groups received the identical MPTP regimen for neurotoxicity induction. On experimental day 15 (post-MPTP), the animals were anesthetized using a gas anesthesia system (2% isoflurane, R500IP, RWD) and then transcardially perfused. The entire brains were immediately collected for subsequent relevant analyses.

### Pole test

To assess bradykinesia and motor coordination, a standardized pole descent test was performed. Mice were placed head-upward at the summit of a vertical pole (55 cm height, 1 cm diameter) covered with medical gauze to increase surface traction. The pole was positioned near the home cage to simulate a natural downward escape behavior. The primary outcome measure was total descent latency, recorded as the time elapsed from initial placement until the animal fully reached the base platform.

### Open field test

Mice were introduced to the periphery of a novel open-field arena (50 × 50 × 40 cm) under low illumination and allowed 10 min of free exploration. Behavior was recorded overhead using Ethovision XT12 software (Noldus, Leesburg, VA) to quantify: (1) center time (10 × 10 cm zone), (2) total ambulatory distance, and (3) entries into defined peripheral (15 cm from walls) and central zones. Arenas were sanitized with 75% ethanol between trials and air-dried. Total distance moved, speed and time served as an index of locomotor activity.

### Immunohistochemistry staining

Following deep anesthesia, mice underwent transcardial perfusion with 0.9% saline followed by 4% paraformaldehyde (PFA). Brains were post-fixed by immersion in cold 4% PFA (24 h) and paraffin-embedded. Coronal sections (5 μm) were deparaffinized, subjected to antigen retrieval, and incubated for 60 min at RT with rabbit anti-tyrosine hydroxylase (TH; 1:500, Affinity). Sections were then treated for 30 min with HRP-conjugated goat anti-rabbit IgG (1:1,000, Abcam), developed with DAB chromogen, and counterstained with hematoxylin. Histological images were captured using an Olympus IX73 inverted microscope.

### Immunofluorescence staining

Colon paraffin sections were deparaffinized in xylene and rehydrated through graded ethanol. Antigen retrieval was performed via microwave heating in EDTA (pH 8.0). After 1 h serum blocking to minimize non-specific binding, sections were incubated overnight at 4°C with primary antibody (anti-ZO-1, DF6442; Affinity Biosciences and anti-Occludin, DF6442; Affinity Biosciences). Following three PBS washes, species-matched fluorescent secondary antibodies were applied. Images were acquired using an Olympus BX61 epifluorescence microscope with fluorophore-specific filter sets.

### Gut microbiota analysis

Fecal DNA extraction utilized the QIAamp PowerFecal DNA kit (Qiagen) following manufacturer specifications, with quantification via Qubit dsDNA HS assay (Invitrogen). The V4 hypervariable region of bacterial 16S rRNA genes was amplified by PCR and sequenced on an Illumina MiSeq platform following Earth Microbiome Project protocols (Caporaso et al., 2012), generating 300-bp paired-end reads. Target sequencing depth was 50,000 reads/sample at Oregon State University’s Center for Quantitative Life Sciences. Bioinformatics processing employed DADA2 in R v3.5 (Callahan et al., 2016), implementing quality trimming, error filtering, read merging, and chimera removal to derive amplicon sequence variants (ASVs). Raw sequencing data were accessible from SRA database (SRA ID: PRJNA1334169).

### Statistical analysis

Data are expressed as mean ± standard deviation (SD). After confirming normality (Shapiro-Wilk test) and homoscedasticity (Brown-Forsythe test), parametric tests (one-way ANOVA with Tukey’s *post-hoc* test) were used for statistical analyses in GraphPad Prism 9.0 (GraphPad Inc., United States). α diversity comparisons among renovation stages and sample sources were assessed by ANOVA with room as a blocking factor. *Post hoc* Tukey’s honest significant difference (HSD) tests were also conducted to correct for multiple comparisons in R-Studio. β diversity significance was determined using ANOSIM tests with 999 permutations. Statistical significance was defined as *p* < 0.05.

## Results

### EA ameliorated motor dysfunction in MPTP mice and reduced the loss of dopaminergic neurons

**FIGURE 1:**
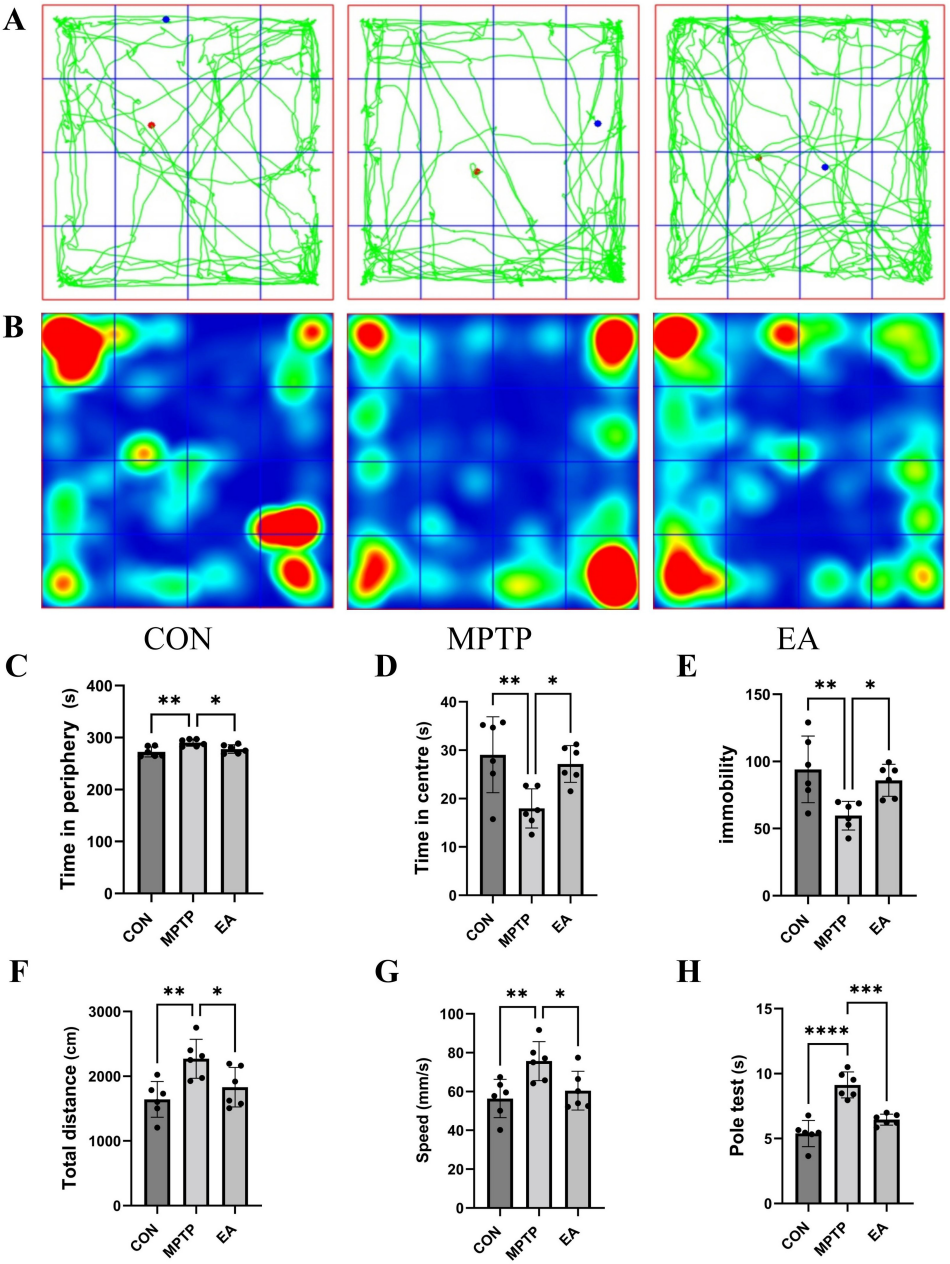
Effects of EA on motor functions in MPTP-induced PD mice. **(A)** Representative trajectories of mice in the open field. **(B)** Cumulative heatmap of time spent in the open field. **(C)** Time spent in the peripheral zone by the three groups of mice. **(D)** Time spent in the central zone by the three groups of mice. **(E)** Immobility time of the three groups of mice. **(F)** Total travel distance of the three groups of mice in the open field. **(G)** Average moving speed of the three groups of mice. **(H)** Performance in the pole test. Data are means ± SD. *n* = 6, *P < 0.05, **P < 0.01, ***P < 0.001, ****P < 0.0001.

**FIGURE 2:**
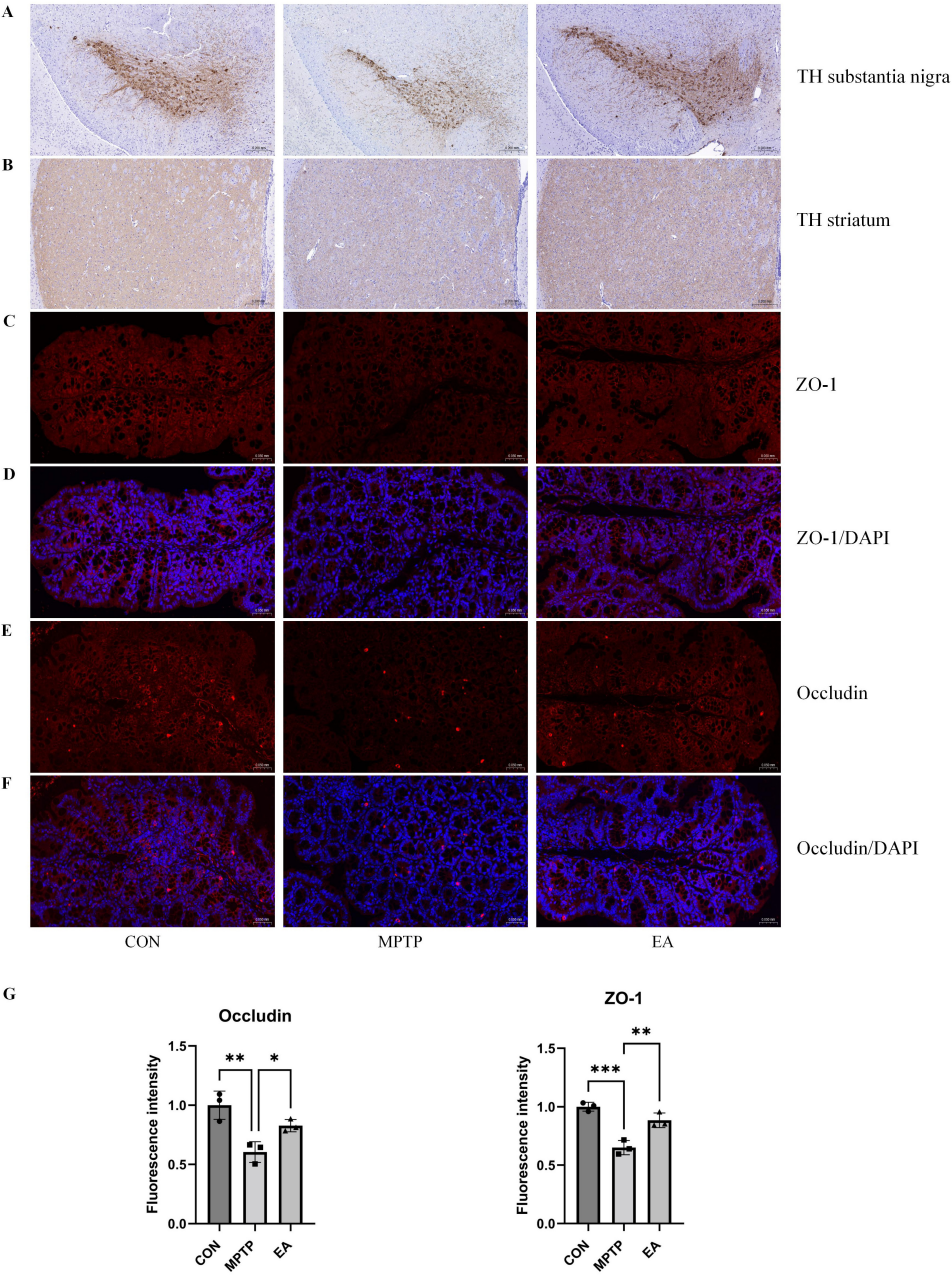
EA restored the MPTP-induced decrease in TH expression in the SN and striatum and alleviated the impairment of colonic intestinal barrier integrity. **(A)** IHC for TH in the SN of brain. **(B)** IHC for TH in the striatum of brain (*n* = 3, Scale bar: 0.2 mm). **(C)** IF for ZO-1 in the colon. **(D)** IF for ZO-1/DAPI in the colon. **(E)** IF for Occludin in the colon. **(F)** IF for Occludin/DAPI in the colon. **(G)** Quantitative fluorescence intensity of Occludin and ZO-1 (*n* = 3, Scale bar: 0.05 mm). *P < 0.05, **P < 0.01, ***P < 0.001.

**FIGURE 3:**
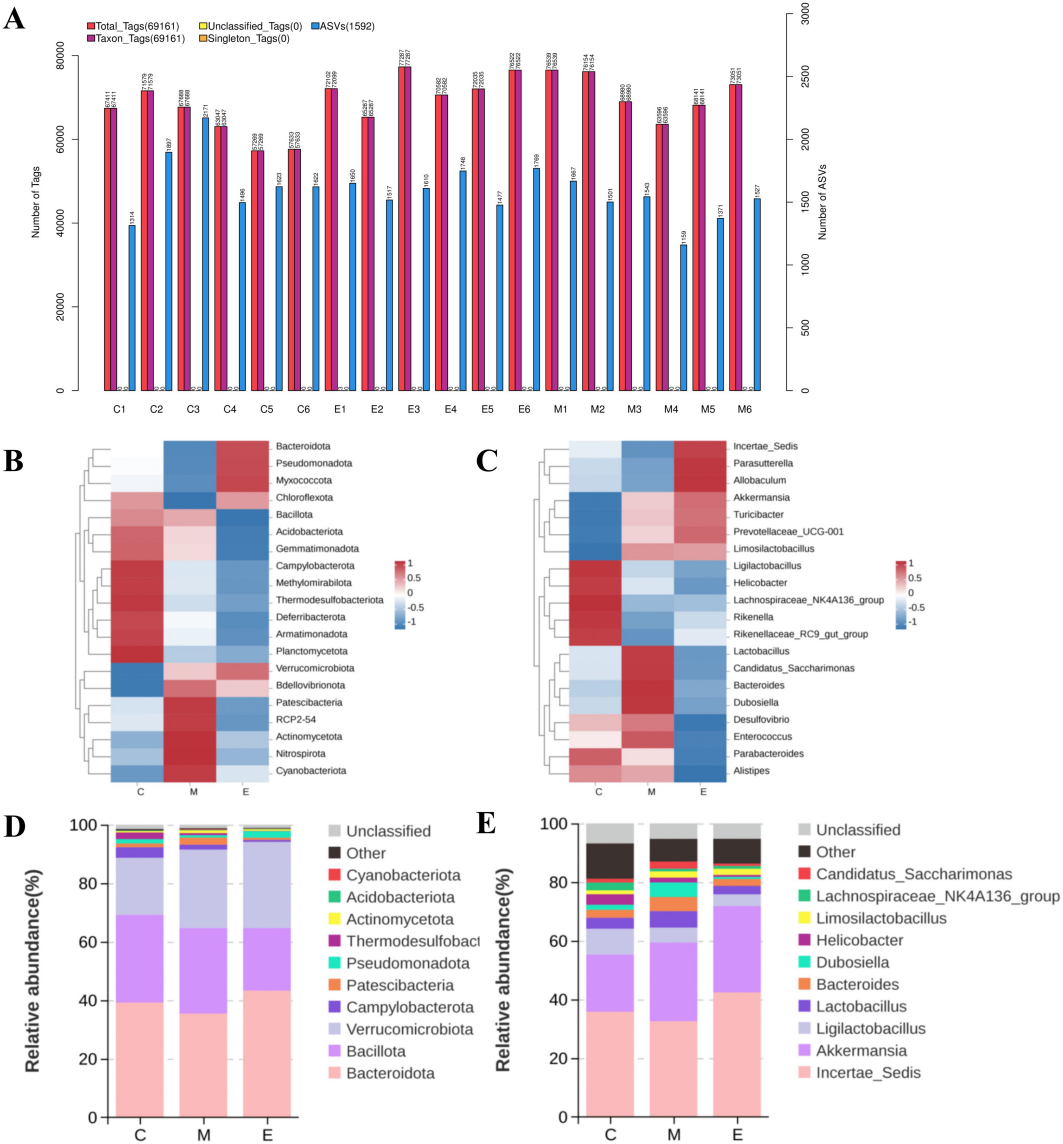
ASV Cluster and Annotation. **(A)** The total tags, unique tags, taxon tags, unclassified tags, and ASV number for each sample. **(B,C)** The heat map of species relative abundance in the phylum and genus level. **(D,E)** The relative abundance of species in the phylum and genus level (top 10). Other indicated the sum of relative abundance beyond the 10 genera.

**FIGURE 4:**
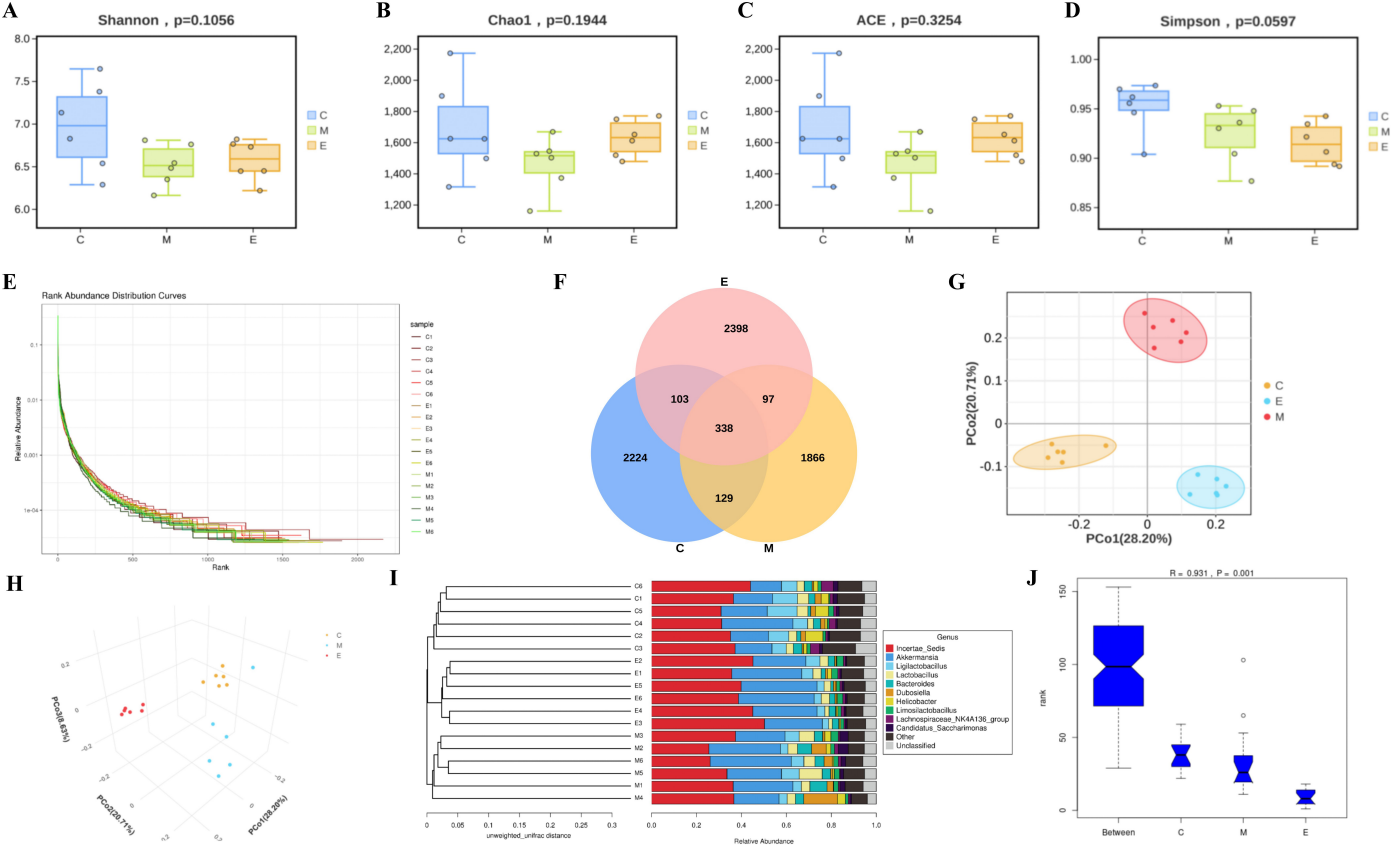
Diversity analysis of gut microbiota. **(A)** Shannon index. **(B)** Chao1 index. **(C)** ACE index. **(D)** Simpson index. **(E)** Rank-abundance curves. **(F)** Venn diagram. **(G,H)** PCoA. **(I)** The UPGMA clustering tree in the genus level. **(J)** ANOSIM analysis in the genus level.

Behavioral assessment revealed significant alterations in locomotor patterns of MPTP-treated mice compared to control mice in the open field test. Specifically: (1) The number of central zone crossings was significantly reduced (Figure 1A); (2) Time spent in the central zone was significantly shortened, while time spent in the peripheral zone was correspondingly prolonged (Figures 1B–D); (3) Immobility time increases in open field (Figure 1E); (4) Both average movement speed and total travel distance were significantly increased (Figures 1F,G). EA intervention effectively reversed these behavioral abnormalities, significantly increasing central zone crossings (Figure 1A), prolonging central zone dwell time (Figures 1B,C), shortening peripheral zone dwell time (Figures 1B,D), shortening immobility time (Figure 1E) and reducing both average speed and total distance traveled (Figures 1F,G). Furthermore, the pole test demonstrated that MPTP mice exhibited a significantly prolonged time to descend from the top to the bottom of the pole compared to controls, and EA treatment significantly shortened this descent time (Figure 1H). Immunohistochemical analysis was further performed to evaluate the neuroprotective effect of EA on dopaminergic neurons. Results showed that MPTP administration caused a significant reduction in the number of dopaminergic neurons in the SNpc and a significant decrease in the density of tyrosine hydroxylase (TH)-positive fibers in the striatum, and EA treatment effectively reversed these MPTP-induced pathological alterations (Figures 2A,B).

### EA ameliorates MPTP-induced intestinal barrier disruption

To assess the impact of MPTP and EA on intestinal barrier integrity, the expression levels of tight junction proteins ZO-1 and Occludin in the colon were evaluated using IF. The results demonstrated that MPTP administration significantly reduced the expression of both ZO-1 and Occludin in colonic tissues. However, subsequent EA treatment effectively reversed this MPTP-induced downregulation, leading to a significant increase in the expression of both proteins compared to the MPTP group (Figures 2C–F).

### Amplicon sequence variant cluster and annotation

The total tags, Singleton tags, taxon tags, unclassified tags, and ASCs number for each sample are shown in Figure 3A. The total numbers for the five indexes above were 1244903, 0, 1244900, 0, and 28662, respectively. Additionally, based on the annotation and abundance information of all subgroups at the phylum and genus level, the genera in the top 20 were shown in the clustering heat map (Figures 3B,C). The top 10 maximum abundance of bacteria in each group in five taxonomic levels (phylum and genus) were selected to generate the column accumulation graph of bacteria relative abundance. The relative abundance of bacteria in the phylum and genus level is shown in Figures 3D,E, respectively. At the phylum level, the 10 most abundant bacterial phyla comprised: *Bacteroidota* (CON: 39.18%, MPTP: 35.43%, EA: 43.16%), *Bacillota* (CON: 29.99%, MPTP: 29.24%, EA: 21.70%), *Verrucomicrobiota* (CON: 19.52%, MPTP: 26.81%, EA: 29.03%), *Campylobacterota* (CON: 3.58%, MPTP: 1.65%, EA: 0.60%), *Patescibacteria* (CON: 1.29%, MPTP: 2.44%, EA: 0.99%), *Pseudomonadota* (CON: 1.47%, MPTP: 0.81%, EA: 1.94%), *Thermodesulfobacteriota* (CON: 2.27%, MPTP: 0.71%, EA: 0.19%), *Actinomycetota* (CON: 0.48%, MPTP: 0.89%, EA: 0.60%), *Acidobacteriota* (CON: 0.16%, MPTP: 0.16%, EA: 0.15%), and *Deferribacterota* (CON: 0.25%, MPTP: 0.12%, EA: 0.08%). At the genus level, the 10 most abundant genera included: *Incertae_Sedis* (CON: 35.81%, MPTP: 32.59%, EA: 42.43%), *Akkermansia* (CON: 19.47%, MPTP: 26.78%, EA: 29.41%), *Ligilactobacillus* (CON: 8.84%, MPTP: 5.10%, EA: 3.98%), *Lactobacillus* (CON: 3.72%, MPTP: 5.60%, EA: 2.88%), *Bacteroides* (CON: 2.73%, MPTP: 4.83%, EA: 2.35%), *Dubosiella* (CON: 1.69%, MPTP: 4.94%, EA: 0.68%), *Helicobacter* (CON: 3.58%, MPTP: 1.65%, EA: 0.67%), *Limosilactobacillus* (CON: 1.32%, MPTP: 2.10%, EA: 2.08%), *Lachnospiraceae_NK4A136_group* (CON: 2.66%, MPTP: 0.91%, EA: 0.97%), and *Candidatus_Saccharimonas* (CON: 1.28%, MPTP: 2.43%, EA: 0.77%).

### Alpha diversity analysis

Analysis of alpha diversity indices revealed no significant differences in Shannon (Figure 4A), Chao1 (Figure 4B), ACE (Figure 4C), or Simpson (Figure 4D) values among the three groups. Rank-abundance curves indicated high taxonomic richness and even species distribution across all three groups (Figure 4E). The Venn diagram depicting ASVs for the three groups is shown in Figure 3F. The three groups shared 338 ASVs. The number of unique ASVs was 2,224 for CON group, 1,866 for MPTP group, and 2,398 for EA group.

### Beta diversity analysis

The results of Principal Coordinates Analysis (PCoA) are presented in Figures 4G,H. Significant differences were observed in the microbiota composition among the three groups (PC1 accounted for 28.20% of the total variation). Furthermore, an unweighted pair group method with arithmetic mean (UPGMA) clustering tree at the genus level is shown in Figure 4I, where *Incertae_Sedis* exhibited the highest relative abundance. Analysis of Similarity (ANOSIM) results confirmed significant differences in the microbiota structure among the three groups (*R* = 0.931, *P* = 0.001) (Figure 4J).

### Linear discriminant analysis effect size analysis

**FIGURE 5:**
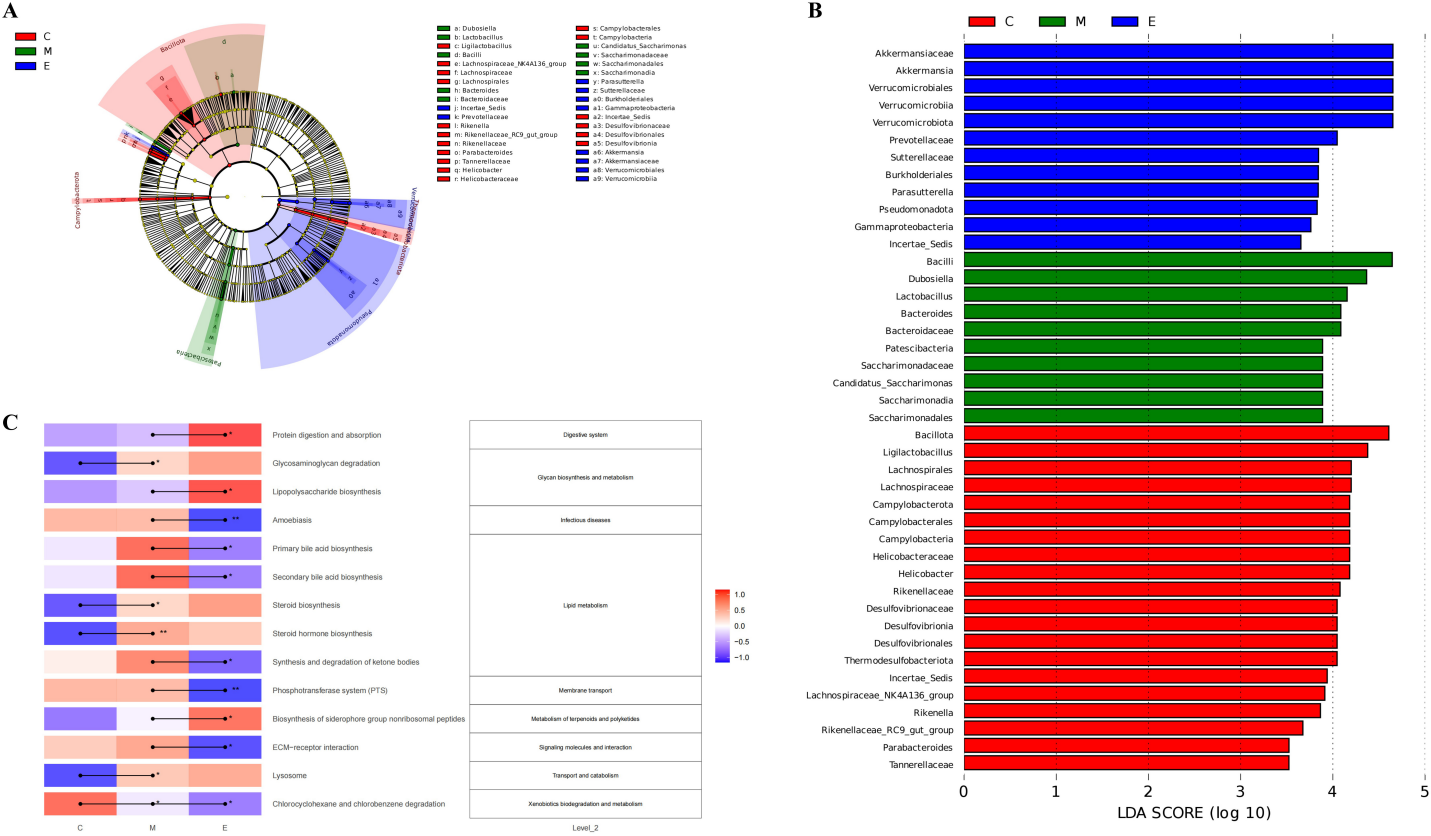
LEfSe analysis and KEGG pathway enrichment. **(A)** Evolutionary branching diagram (LDA > 3.5). **(B)** LDA value distribution histogram (LDA > 3.5). **(C)** Differential KEGG pathway.

LEfSe analysis was employed to identify statistically significant discriminant biomarkers among the groups. As shown in Figure 5B, significantly enriched taxa in the CON group included the phylum *Bacillota*, the genus *Ligilactobacillus*, and the order *Lachnospirales*, among others. In the MPTP group, significantly enriched taxa comprised the class *Bacilli*, the genus *Dubosiella*, and the genus *Lactobacillus*. The EA group exhibited significant enrichment in the family *Akkermansiaceae*, the genus *Akkermansia*, and the order *Verrucomicrobiales*. The phylogenetic tree illustrating these results is presented in Figure 5A.

### EA changes the functionality of the gut microbiota in MPTP-induced PD mice

Fourteen Kyoto Encyclopedia of Genes and Genomes (KEGG) pathways (level 3) were significantly altered in either the model group or the EA group (Figure 5C). Many of these predicted functional differences involved lipid metabolism pathways. Based on level 2 KEGG pathway analysis, the MPTP group exhibited disturbances in Transport and catabolism, Lipid metabolism, and Signaling molecules and interaction. EA treatment affected 10 signaling pathways, three of which belonged to the lipid metabolism category. Consequently, we further analyzed the Transport and catabolism, Lipid metabolism, and Signaling molecules and interaction based on level 3 KEGG classifications. The results revealed that four pathways were significantly disturbed in the model group: Glycosaminoglycan degradation, Steroid bile acid biosynthesis, Steroid hormone biosynthesis, Lysosome, and Chlorocyclohexane and chlorobenzene degradation. EA treatment affected 10 signaling pathways. Among these, Primary bile acid biosynthesis, Secondary bile acid biosynthesis, Steroid bile acid biosynthesis, and Steroid hormone biosynthesis are all known to be associated with PD pathogenesis (Tong et al., 2024).

### Co-occurrence networks

**FIGURE 6:**
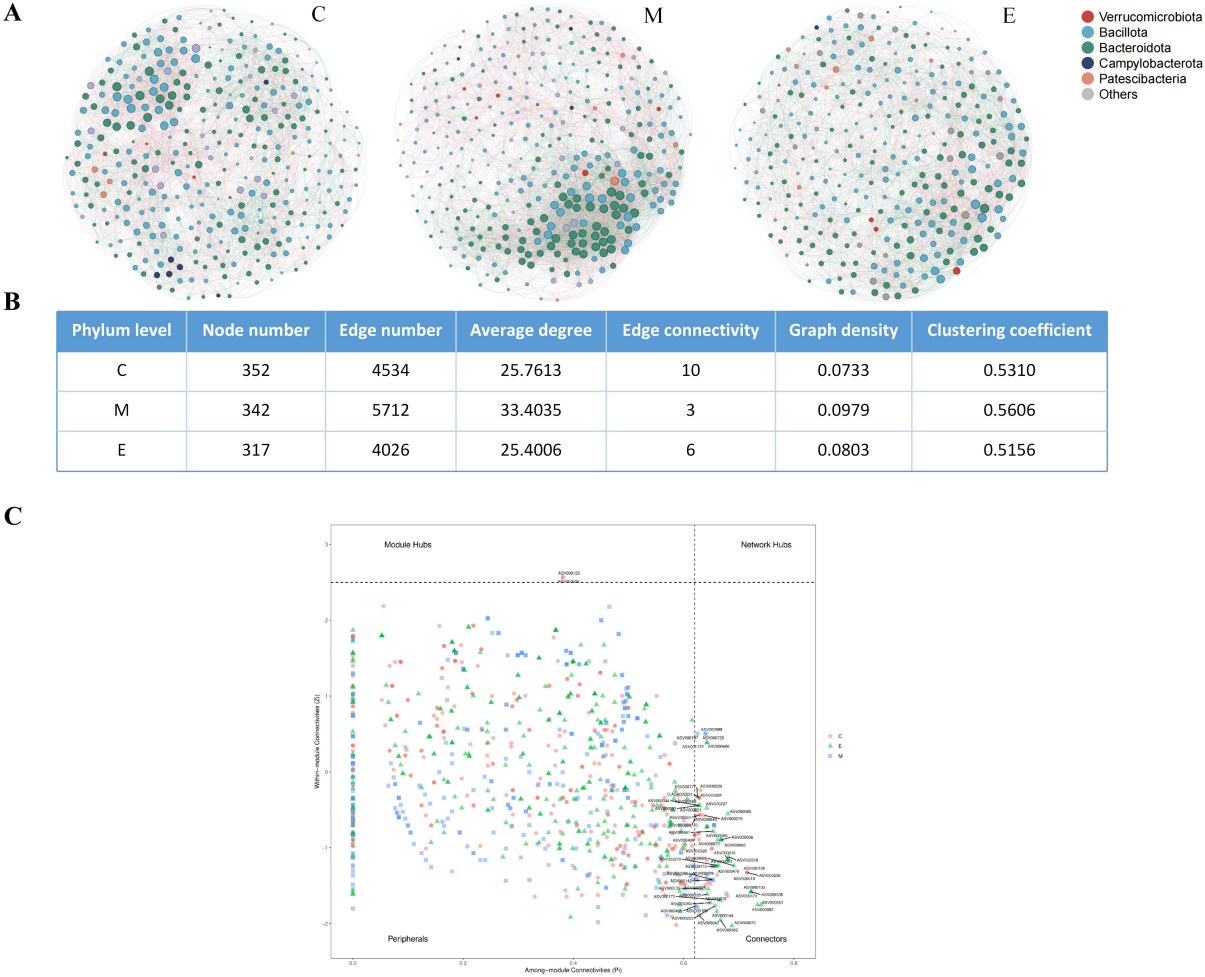
Co-occurrence network analysis. **(A)** Co-occurrence network of three groups. **(B)** Network topology parameter. **(C)** Zi-Pi plot.

Potential keystone taxa were identified using values of within-module connectivity (Zi) and among-module connectivity (Pi) for each ASV. Nodes were classified into four categories: module hubs (Zi > 2.5 and Pi ≤ 0.62), network hubs (Zi > 2.5 and Pi > 0.62), connectors (Zi ≤ 2.5 and Pi > 0.62), and peripherals. Due to their topological importance within the network, network hubs, module hubs, and connectors were designated as keystone taxa. Compared to the control group network, the MPTP group network exhibited higher values for Node number, Edge number, Average degree, Graph density, and Clustering coefficient, but lower Edge connectivity (Figures 6A,B). EA treatment reversed these effects for all metrics except Node number. Notably, the detected module hubs (ASV000123 and ASV000325) and the majority of connectors were low-abundance taxa, suggesting that bacteria with lower relative abundance play regulatory roles within the microbial co-occurrence network (Figure 6C)

### Associations between gut microbiota and motor function in PD mice following EA treatment

**FIGURE 7:**
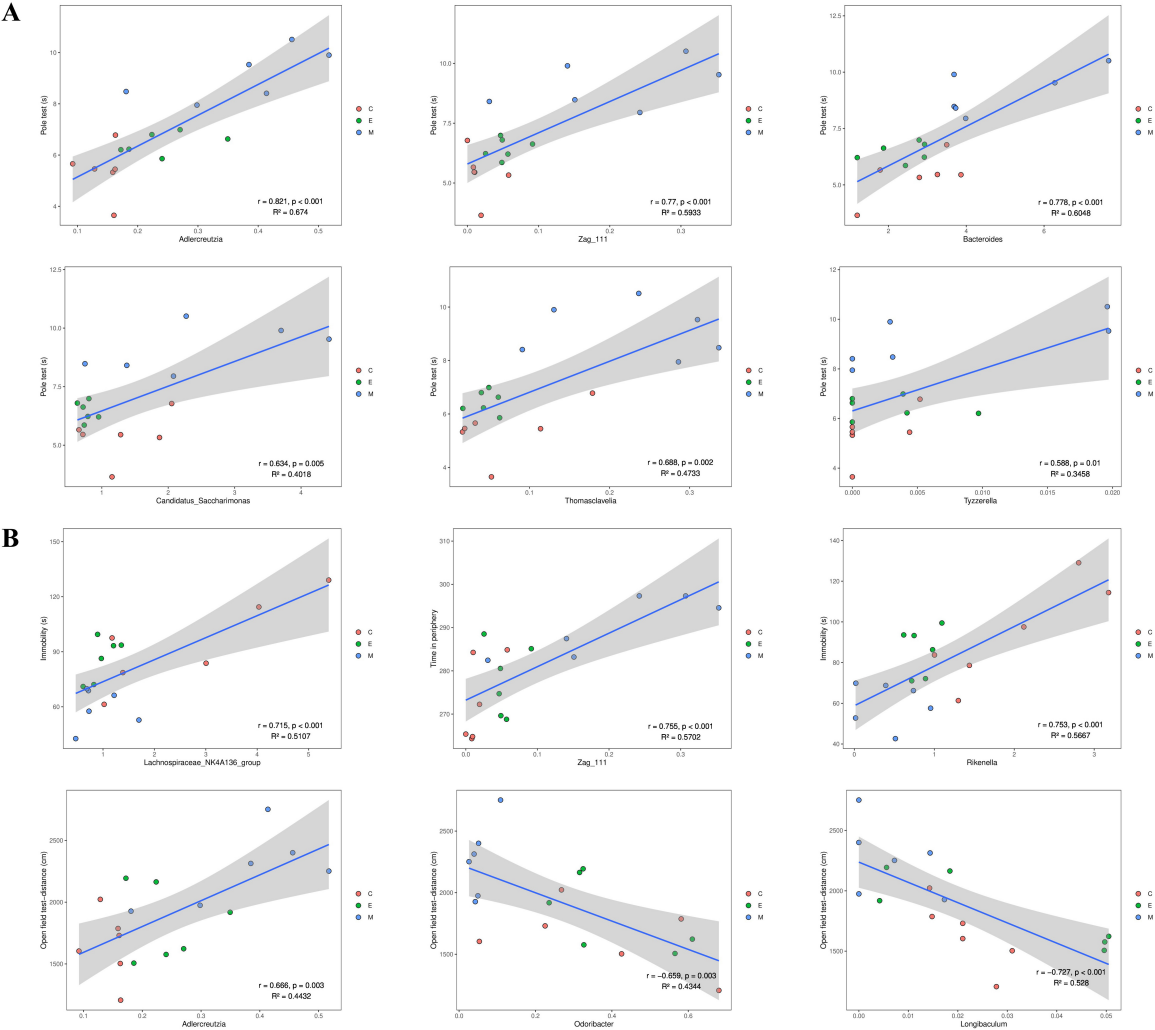
Correlation analysis of gut genera and behavior. Spearman’s correlation analyses between the relative abundance of gut microbiota at the genus level and the value in **(A)** pole test or **(B)** open field test, respectively. Significant correlations were determined based on |Spearman r| > 0.5 and *p* < 0.5 (Hu et al., 2024).

To investigate whether gut microbiota correlates with clinical features of PD and to determine if significant alterations in both gut microbiota and PD clinical characteristics following EA treatment are interrelated, we performed Spearman correlation analysis between the gut microbiota (represented by 96 genera shared across all three groups; see Supplementary Table 1) and motor function. Motor function was assessed using representative parameters of behavioral deficits exhibited by the model group in the balance beam and open field tests. Figure 7 presents representative correlations selected based on absolute Spearman r values. *Candidatus Saccharimonas*, *Thomasclavelia*, *Bacteroides*, *Adlercreutzia*, *Zag_111*, and *Tyzzerella* showed significant associations with pole test performance, while *Lachnospiraceae_NK4A136_group*, *Zag_111*, *Rikenella*, *Adlercreutzia*, *Odoribacter*, and *Longibaculum* correlated with open field test parameters.

## Discussion

PD is a common neurodegenerative disorder characterized by cardinal motor symptoms including bradykinesia, resting tremor, muscle rigidity, and postural instability, alongside non-motor symptoms such as depression and sleep disturbances (Ao, 2022). Accumulating clinical evidence has demonstrated that EA stimulation can alleviate both motor and non-motor symptoms in PD patients (Ao, 2022). However, the underlying mechanisms through which EA ameliorates motor dysfunction remain elusive. As MPTP is the only neurotoxin known to induce a clinical syndrome indistinguishable from PD in both humans and non-human primates, it was employed to establish the PD model in this study (Jackson-Lewis and Przedborski, 2007). PD was modeled in mice through intraperitoneal administration of MPTP (30 mg/kg daily for 5 consecutive days), an established protocol documented in prior literature (Sun et al., 2018); consistent with these reports, MPTP-treated mice exhibited motor dysfunction alongside degeneration of SN dopaminergic neurons and reduced TH expression. In behavioral assessments, while MPTP group mice showed significantly prolonged descent time in the pole test versus controls—ameliorated by EA treatment—they paradoxically demonstrated increased locomotion velocity and total distance traveled in the open field test, with EA significantly reducing both hyperactivity parameters. The specific mechanism underlying this phenomenon has not yet been systematically elucidated, although similar observations have been reported in numerous studies (Sedelis et al., 2001). The subacute dosing regimen employed in this study represents the upper limit of conventional dosage used in this type of PD model (Jackson-Lewis and Przedborski, 2007). Previous research has suggested that extremely high doses of MPTP may induce hyperactive behavioral phenotypes in experimental animals by affecting non-dopaminergic pathways such as the serotonergic system (Chia et al., 1996; Chia et al., 1999). Additionally, potential stress factors inherent in the experimental procedure warrant attention: the transfer of animals from the housing room to the testing room prior to behavioral assessments may elicit an acute stress response. Studies have demonstrated that stress can significantly activate the dopaminergic system—for instance, dopamine release in the striatum, nucleus accumbens, and medial prefrontal cortex of rats increases markedly under stress conditions (Abercrombie et al., 1989). In animals with pre-existing nigrostriatal system impairment, such stress-induced neurochemical changes may overload compensatory mechanisms, leading to transient paradoxical enhancement of motor function (Zigmond and Stricker, 1989). In summary, the observed hyperactivity in the open field test among MPTP-treated mice in this study may be attributed to the combined effects of high-dose MPTP and stress responses triggered by experimental handling and transportation.

The human gut microbiota exhibits high diversity and complex structural organization, maintaining metabolic, immune, and endocrine homeostasis under physiological conditions (Khlevner et al., 2018). Substantial evidence implicates gut dysbiosis in the pathogenesis of multiple peripheral and central nervous system disorders, with the bidirectional gut-brain axis serving as a critical communication pathway regulating these systemic interactions (Al Omran and Aziz, 2014; Khlevner et al., 2018). Notably, emerging research highlights significant compositional alterations in the gut microbiota of PD patients compared to healthy individuals (Zhang X. et al., 2023), wherein gut microbes may contribute to α-SYN aggregation and PD pathogenesis (Sampson et al., 2016). Landmark evidence demonstrates that fecal microbiota transplantation from PD patients exacerbates neuroinflammation and neurodegeneration in PD mouse models, underscoring the microbiota’s crucial role in disease progression (Yang et al., 2024).

In this study, 16S rRNA gene sequencing was employed to investigate alterations in the gut microbiota of PD model mice. While no significant differences in α-diversity indices (reflecting species richness, evenness, or diversity) were observed among the three experimental groups, β-diversity analysis revealed significant compositional divergence, indicating distinct overall community structures. This demonstrates that EA modulated MPTP-induced shifts in microbial community architecture. Linear discriminant analysis (LDA) identified *Dubosiella* as the genus most substantially impacted by MPTP treatment. Classified within the phylum Bacillota (formerly Firmicutes) and family *Erysipelotrichaceae*, *Dubosiella* exhibited increased abundance in MPTP mice—consistent with prior reports (Pu et al., 2023; Shao et al., 2025). Furthermore, its elevation correlated positively with phosphorylated α-SYN in the brain and colon, and negatively with TH expression in the brain (Pu et al., 2023). These effects may relate to *Dubosiella newyorkensis*’ capacity to mitigate oxidative stress, improve vascular endothelial function, redistribute gut microbiota, and produce isovaleric acid (IAA) (Liu et al., 2023; Shao et al., 2025). Although current evidence regarding *Dubosiella*’s role in PD remains limited, extant studies suggest potential protective properties (Jiang et al., 2023); however, discrepancies exist as one study reported decreased *Dubosiella* abundance in rotenone-induced PD models—reversed by EA—likely attributable to variations in modeling methods, EA parameters, and acupoint selection (Hu et al., 2024). Furthermore, *Lactobacillus*, *Enterococcus*, *Desulfovibrio*, *Bacteroides*, and *Candidatus Saccharimonas* exhibited abundance changes consistent with *Dubosiella*. Among these, *Lactobacillus* (phylum Firmicutes) represents the most extensively studied genus and a key probiotic in the gut microbiome; these commensal bacteria engage in intra-species communication and cross-talk with the intestinal epithelium to maintain barrier integrity, enhance mucosal defense, and modulate host immune responses (Martín et al., 2019). Mechanistically, lactobacilli exert antimicrobial effects through competitive exclusion of opportunistic pathogens, inhibition of epithelial adhesion, and direct pathogen suppression via production of lactic acid, acetate, propionate, bacteriocins, and reactive oxygen species (Dempsey and Corr, 2022). Clinically, 12-week supplementation with *L. plantarum* PS128 significantly improved Unified Parkinson’s Disease Rating Scale (UPDRS) motor scores and quality of life in PD patients, suggesting therapeutic potential as an adjunct treatment (Lu et al., 2021). Preclinically, oral administration of *L. plantarum* PS128 attenuated MPTP-induced elevations in corticosterone, nigrostriatal dopaminergic neuron loss, and striatal dopamine depletion while increasing dopamine transporter (DAT) expression and norepinephrine secretion in murine models (Liao et al., 2019; Liao et al., 2020). In rotenone-induced PD models, PS128 ameliorated motor dysfunction and conferred neuroprotection by modulating gut microbiota, suppressing the miR-155-5p/SOCS1 pathway, and reducing microglial activation—though a recent study reported electroacupuncture at ST25 acupoint reversed rotenone-induced *Lactobacillus* depletion, indicating discrepant outcomes may reflect strain-specific effects or variations in therapeutic parameters (Lee et al., 2023; Hu et al., 2024).

Within these genera, *Bacteroides* (phylum Bacteroidetes) emerges as another beneficial microbe—a core colonizer of the human colon constituting a major proportion of the gut bacterial community (Kim et al., 2017). These Gram-negative obligate anaerobes perform multifaceted roles as keystone participants in maintaining microbial food webs (Wexler, 2007). Functioning as proven commensals, mutualists, and beneficial organisms, they serve as “suppliers” providing essential functions for both host and neighboring microbes while conferring diverse health benefits. Specifically, *Bacteroides* enhances glucose metabolism, degrades complex plant polysaccharides, and produces neuroactive short-chain fatty acids (SCFAs) and vitamins crucial for intestinal health (Kovatcheva-Datchary et al., 2015). Beyond these symbiotic effects, their metabolites modulate dopaminergic synaptic activity; Hartstra et al. recently demonstrated that fecal microbiota transplantation (FMT) of *Bacteroides uniformis* increases striatal dopamine transporter (DAT) binding (Hartstra et al., 2020). As a presynaptic membrane protein regulating synaptic and extracellular dopamine levels, DAT critically governs dopaminergic signaling. Dopamine recycled via DAT into presynaptic vesicles enables subsequent neurotransmitter release. Paradoxically, elevated *Bacteroides* abundance correlates positively with plasma pro-inflammatory cytokine TNF-α in PD patients—likely mediated by lipopolysaccharide (LPS)-stimulated TNF-α secretion from macrophages and monocytes (Delahooke et al., 1995; Lin et al., 2019). Consistently, recent studies detected elevated fecal inflammatory markers (e.g., IL-1, CXCL8) in PD patients versus controls (Houser et al., 2018). Collectively, the upregulated abundance of *Lactobacillus* and *Bacteroides* observed in our study may represent a compensatory protective response within the murine host system.

In contrast to the protective roles of *Lactobacillus* and *Bacteroides*, elevated abundances of *Enterococcus* and *Desulfovibrio* exerted detrimental effects. Levodopa (L-dopa) serves as the cornerstone dopamine-replacement therapy for PD, typically co-administered with decarboxylase inhibitors to enhance bioavailability by preventing peripheral metabolism (Maini Rekdal et al., 2019). However, L-dopa/decarboxylase inhibitor combinations prove ineffective in some patients, with diminishing efficacy over time necessitating frequent dosage adjustments (Maini Rekdal et al., 2019). Critically, ileal tyrosine decarboxylase (TyrDC) gene abundance in PD patients correlates positively with L-dopa dosage but negatively with plasma L-dopa levels (van Kessel et al., 2019). Mechanistically, *Enterococcus faecalis*—a primary source of TyrDC—decarboxylates both its preferred substrate tyrosine (structurally analogous to L-dopa differing by a single hydroxyl group) and L-dopa itself, as confirmed by genetic and biochemical assays (Brüssow, 2020). Recent studies demonstrate that *E. faecalis* decarboxylates L-dopa independently of carbidopa inhibition (Maini Rekdal et al., 2019). Consequently, PD patients harboring high *E. faecalis* burdens exhibit reduced peak serum L-dopa concentrations; this microbial metabolism peripherally converts L-dopa to dopamine, which cannot cross the blood-brain barrier, thereby decreasing neuronal L-dopa uptake while elevating systemic dopamine and its derivative m-tyramine—contributing to adverse effects (Jameson and Hsiao, 2019; Maini Rekdal et al., 2019). Thus, *E. faecalis* abundance and TyrDC expression predict interindividual variations in L-dopa metabolism within complex gut microbiomes, suggesting that inhibiting this bacterium could enhance L-dopa bioavailability. Notably, our study revealed EA reduced *E. faecalis* abundance (Supplementary Figure 1), indicating EA may curtail peripheral L-dopa metabolism, potentially explaining the superior clinical efficacy and dose-sparing effects observed when acupuncture is combined with L-dopa therapy.

Sulfate-reducing bacteria, primarily represented in humans by the genus *Desulfovibrio*, are anaerobic microorganisms that generate energy through dissimilatory sulfate reduction, producing substantial hydrogen sulfide (H2S) (Willis et al., 1997; Loubinoux et al., 2002). Case-control studies demonstrate increased relative abundance of *Desulfovibrio* in PD gut microbiomes, with recent evidence confirming its strong disease association (Lin et al., 2018). Specifically, fecal *Desulfovibrio* loads correlate positively with PD severity and constipation—an early PD symptom—while *Desulfovibrio*-specific [FeFe]-hydrogenase genes are consistently detected in PD samples, suggesting its potential as a PD progression biomarker (Murros et al., 2021; Fan et al., 2022). Crucially, *Desulfovibrio* contributes to PD pathogenesis through dual mechanisms: (1) Sulfate respiration generates H2S which, beyond physiological signaling roles, induces mitochondrial cytochrome c release at neurotoxic concentrations; cytosolic cytochrome c peroxidase activity promotes α-SYN oligomerization, while H2S concurrently disrupts iron homeostasis by increasing cytosolic iron—a known inducer of α-SYN fibrillation (Calvert et al., 2010; Carbonero et al., 2012). (2) Periplasmic [FeFe]-hydrogenase (ubiquitous in *Desulfovibrio*) reduces Fe^3+^ to Fe^2+^, forming Fe_3_O_4_ nanoparticles capable of crossing the blood-brain barrier and accelerating cerebral α-SYN aggregation (Park et al., 2008). Additionally, *Desulfovibrio*-derived lipopolysaccharides alter macrophage miRNA expression, triggering pro-inflammatory cascades (Murros, 2021). Consistent with our findings, rotenone-induced PD models exhibit *Desulfovibrio* overgrowth—reversed by fecal microbiota transplantation from healthy mice (Zhao et al., 2021). Notably, EA may ameliorate this pathology by reducing *Desulfovibrio* abundance.

Previous studies have established that disorders of lipid metabolism are prevalent in PD patients (Galper et al., 2022). Animal research further confirms a close association between lipid metabolism dysregulation and both α-SYN aggregation and neuroinflammation(Tong et al., 2024). KEGG pathway analysis results indicate that lipid metabolism plays a significant role in our study. The relative abundance of *Allobaculum* species correlates with fatty acid metabolism, high-fat diet, and aging (Lichtenstein and Kersten, 2010; Cox et al., 2014; Fernández-Hernando and Suárez, 2020). Research identifies ANGPTL4 as a circulating mediator linking the gut microbiota to fat deposition; it acts as a crucial regulator of triglyceride metabolism by inhibiting lipoprotein lipase and pancreatic lipase (Alex et al., 2014; Aryal et al., 2019) (Chia et al., 1999; Sedelis et al., 2001). Notably, the abundance of *Allobaculum* is positively correlated with ANGPTL4 expression levels. In mouse models of metabolic syndrome, *Allobaculum* has been shown to protect against and ameliorate symptoms, with its abundance positively correlating with ileal levels of RORγT and IL-17 (Cox et al., 2014). Furthermore, Herrmann et al. reported that *Allobaculum* is an active glucose-utilizing bacterium capable of producing lactate and butyrate (Herrmann et al., 2017; Pujo et al., 2021). Therefore, EA may improve lipid metabolism and mitigate MPTP-induced effects in mice by upregulating the abundance of *Allobaculum* and promoting lactate secretion. Another genus exhibiting a similar trend in abundance change to *Allobaculum* is *Parasutterella*. *Parasutterella* remains a relatively novel taxonomic unit, and the available literature is limited. One study on the association between gut microbiota and diet-induced obesity reported decreased abundance of *Parasutterella* in diet-induced obese mice, while its abundance significantly increased in control groups and in mice switched from a high-fat diet back to normal chow (Zhang et al., 2012). Another study found that upregulated expression of *Parasutterella* in the submucosa was closely linked to Crohn’s disease and hypertriglyceridemia-associated acute necrotizing pancreatitis (Chiodini et al., 2015; Huang et al., 2017). This suggests *Parasutterella* may be associated with lipid metabolism and Crohn’s disease; however, this appears inconsistent with our findings of decreased *Parasutterella* abundance and increased gut inflammation in MPTP mice, warranting further investigation into its functional role. Another genus implicated in lipid metabolism is *Akkermansia*, a potential next-generation probiotic associated with host mucus turnover. It degrades mucin, releasing sulfur-containing amino acids utilized by other gut microbes for sulfur metabolism (Hertel et al., 2019). In our results, *Akkermansia muciniphila* was the primary contributor to the genus *Akkermansia*’s abundance. Studies indicate that *A. muciniphila* modulates the gut FXR-FGF15 axis, remodels bile acid composition, and reduces the levels of secondary bile acids, including deoxycholic acid and lithocholic acid, in the cecum and liver (Wu et al., 2023). Moreover, *Akkermansia* has been shown to effectively ameliorate conditions like ulcerative colitis (Wexler, 2007), obesity (Kovatcheva-Datchary et al., 2015), and amyotrophic lateral sclerosis (Hartstra et al., 2020) by protecting intestinal barrier function and reducing levels of colonic inflammatory cytokines (TNF-α, IL-1β, IL-6) (Bian et al., 2019). Previous research confirmed an increased relative abundance of *Akkermansia* in PD patients (Keshavarzian et al., 2015; Lee et al., 2020; Vascellari et al., 2021) (Chiodini et al., 2015; Liu et al., 2021; Choi et al., 2022; Kim et al., 2022; Lam et al., 2022; Tan et al., 2022), which aligns with our findings. During mucin degradation, *A. muciniphila* produces acetate and propionate, which serve as substrates for other bacteria and the host and confer protective effects (Derrien et al., 2004; Lukovac et al., 2014). For instance, butyrate protects against dopaminergic neuron loss and motor dysfunction in PD mouse models by stimulating glucagon-like peptide 1 (GLP-1) (Liu et al., 2017). Additionally, butyrate can reduce blood-brain barrier permeability, microglial activation, and PD-associated depressive symptoms (Xie et al., 2022). Furthermore, propionate and butyrate suppress neuroinflammation by inhibiting cytokine storms and viral pathogenicity (McCarville et al., 2020; Majumdar et al., 2023). Nevertheless, whether the increased relative abundance of *Akkermansia* is beneficial or detrimental remains controversial (Chiantera et al., 2023). Other studies propose that excessive enrichment of *Akkermansia* may disrupt mucin degradation processes, thereby impairing intestinal barrier function and inducing the secretion of inflammatory factors (Khan et al., 2020). Research indicates that *Akkermansia* increases significantly in the early stages of PD. Its effects may be dose-dependent and could exert differential roles at various stages of PD, closely linked to the regulation of neuroinflammation. In our study, the abundance of *A. muciniphila* significantly increased in both the MPTP and EA groups (Supplementary Figure 1), suggesting that its protective role predominates in our experimental context. However, the above findings are primarily speculative, based on our sequencing data and previous literature. The specific causal relationships require further experimental validation.

Zi-Pi plot and correlation analyses suggest that low-abundance microbial communities may play a role in the development of PD, though their low abundance makes them prone to being overlooked during initial screening. *Adlercreutzia* has been previously reported as a genus capable of producing equol in human feces (Maruo et al., 2008). Studies indicate that *Adlercreutzia* is significantly reduced in PD mice, and its abundance shows a negative correlation with microglial counts in the SNpc and striatum, while exhibiting a positive correlation with taurine levels. This suggests *Adlercreutzia* may exert anti-neuroinflammatory effects in PD mice. Subedi et al. further confirmed that *Adlercreutzia*, through equol production, exhibits potent antioxidant and anti-inflammatory properties, which were demonstrated to alleviate microglia-mediated neuroinflammation by inhibiting NF-κB activation (Subedi et al., 2017). Contrary to these previous reports, our findings revealed a significant *increase* in *Adlercreutzia* abundance in the MPTP group, which subsequently decreased following EA treatment. Therefore, we speculate that the elevated abundance of *Adlercreutzia* in MPTP mouse may represent a compensatory response aimed at counteracting microglial activation and reducing associated neuroinflammation. Notably, genera including *Adlercreutzia* were significantly correlated with behavioral performance. While this strong association is highly suggestive of their participation in the motor function improvements mediated by electro-acupuncture, the definitive causality remains to be determined.

As previously discussed, multiple microbial genera act as “suppliers,” providing energy substrates and other essential materials to the host and neighboring microbes, serving as key participants in maintaining the gut microbial food web. Consequently, the connections between different genera can significantly influence host health and the development of disease. This study found that MPTP treatment and EA therapy significantly altered the topology of the gut microbial co-occurrence network. Compared to the control group, MPTP treatment reduced the number of microbial species participating in the network (Node number) but significantly increased the number of significant interactions between microbes (Edge number), the average degree (representing the number of interaction partners per species), Graph density (reflecting the tightness of community interactions), and the clustering coefficient (characterizing the local modular strength). This indicates that MPTP enhanced the interaction intensity and local clustering within the microbial community. However, this enhanced connectivity was accompanied by reduced edge connectivity, suggesting decreased network stability induced by MPTP. EA treatment further reduced the number of co-occurring species (Node number) while simultaneously reversing the MPTP-induced enhancement of network connectivity, evidenced by significant decreases in Edge number, Average degree, Graph density, and Clustering coefficient. Notably, EA significantly increased edge connectivity, thereby enhancing the overall stability of the network and improving the microbial community’s resilience to perturbation. Collectively, integrating these findings with the β-diversity results, MPTP altered microbial composition and promoted microbial interactions, yet resulted in a network state characterized by heightened connectivity but diminished stability. In contrast, EA therapy restored a functional state closer to the control group by suppressing excessive interactions, streamlining connections, and enhancing network stability.

This study reveals that the MPTP-induced PD model exhibits a unique hyperkinetic phenotype concurrent with dysbiosis of the gut microbial co-occurrence network, characterized by increased connection complexity but reduced stability. Based on the above results and previous literature, we speculate that EA treatment may ameliorate this condition by remodeling the microbial structure—significantly suppressing pro-inflammatory/neurotoxic genera (such as *Desulfovibrio* and *Enterococcus*) while elevating the abundance of protective genera (e.g., *Allobaculum*)—thereby restoring the topological stability of the microbial network. This reconstruction of network homeostasis drives a rebalancing of microbial functional modules (e.g., regulating lipid and sulfur metabolism, mitigating neuroinflammation, and inhibiting peripheral utilization of levodopa). Ultimately, these changes improve dopaminergic neuronal function and reverse motor deficits via the gut-brain axis pathway. It is important to note that these findings warrant additional investigation to establish causality. In the future, we will conduct relevant experiments to validate the findings of this study. First, we will perform FMT to directly observe whether recipient mice transplanted with the “EA microbiota” can replicate the neuroprotective phenotypes observed in this study. Second, we will utilize targeted metabolomics technology to detect the levels of microbial metabolites, such as short-chain fatty acids, in the serum and brain tissue of mice. We also plan to orally supplement key metabolites to validate their function in animal models. Finally, we will attempt to re-establish the PD model under germ-free conditions to ultimately determine whether the gut microbiota is a necessary condition for the efficacy of EA. This study is the first to observe the effects of 100 Hz EA at GV20 and GV14 on gut microbiota alterations in MPTP-induced PD mice. We discovered that EA regulated several genera and species implicated in PD pathogenesis, including *Desulfovibrio*, *Allobaculum*, *Akkermansia muciniphila*, *Enterococcus faecalis* and so on. These findings provide a theoretical basis for developing precise electroacupuncture treatment strategies based on gut microbiota modulation.

## Data Availability

The datasets presented in this study can be found in online repositories (SRA ID: PRJNA1334169; https://www.ncbi.nlm.nih.gov/sra/PRJNA1334169).
