# Supplementary material for: Electroacupuncture exerts neuroprotective effects and alters gut microbiota in a MPTP-induced mouse model of Parkinson’s disease
Source: Front Neurosci. 2026 Jan 14;19:1702912. doi: 10.3389/fnins.2025.1702912 (PMC12847003; doi:10.3389/fnins.2025.1702912)
Supplement: Supplementary file 1 [file Data_Sheet_1.docx]

**EA modulates the species abundance changes induced by MPTP.**

EA treatment reversed the MPTP-induced increase in the abundance of *Enterococcus faecalis* (Fig. S1A) and increased he abundance of *Akkermansia muciniphila* (Fig. S1B).


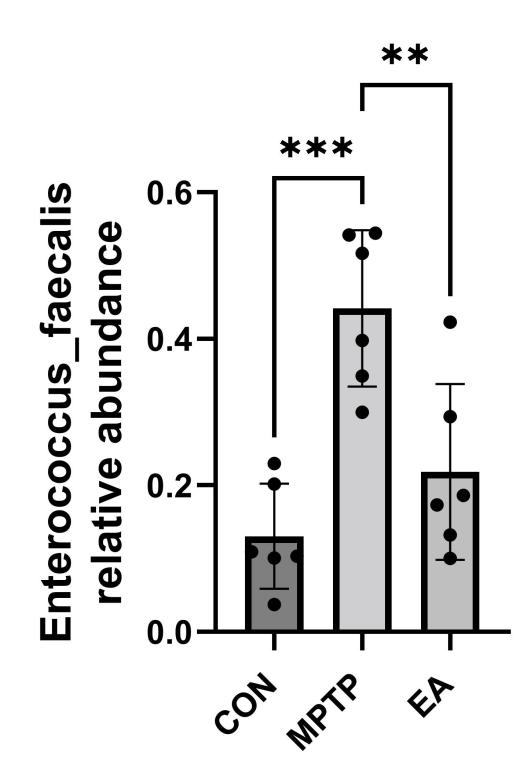

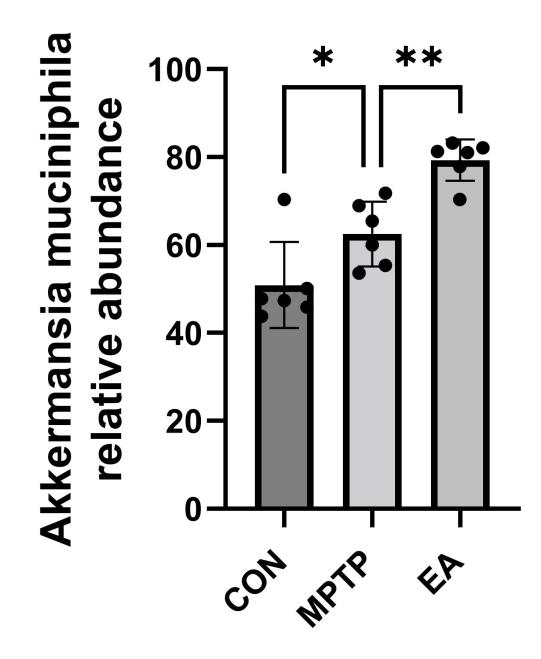


**A**

**B**

Fig. S1 The effect of EA on the abundance of *Enterococcus faecalis* and *Akkermansia muciniphila*. (A) *Enterococcus faecalis*. (B) *Akkermansia muciniphila.* Data are means ± SD. n = 6, **P* < 0.05, ***P* < 0.01, ****P* < 0.001.
